# Supplementary material for: Human IgMhiCD300a+ B Cells Are Circulating Marginal Zone Memory B Cells That Respond to Pneumococcal Polysaccharides and Their Frequency Is Decreased in People Living with HIV
Source: Int J Mol Sci. 2023 Sep 6;24(18):13754. doi: 10.3390/ijms241813754 (PMC10530418; doi:10.3390/ijms241813754)
Supplement: Supplementary file 1 [file ijms-24-13754-s001.zip › ijms-2554225-supplementary.pdf]

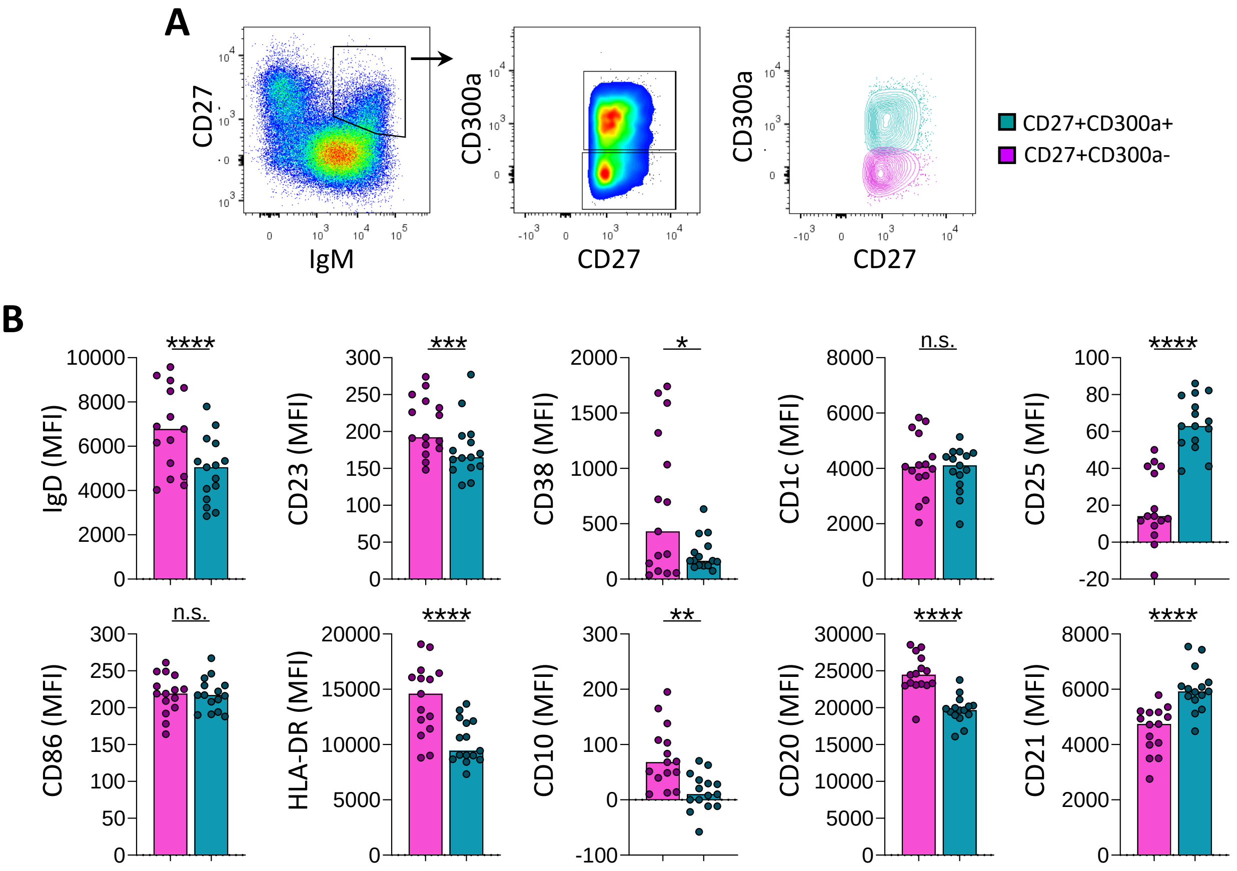

Supplementary Figure S1. Phenotypical characterization of IgM^{hi}CD27+300a+ and IgM^{hi}CD27+300a-. (A) Pseudocolor plots of concatenated peripheral CD19+ B cells from healthy donors in which IgM^{hi}CD27+ were analyzed based on CD300a expression. CD300a+ (green) and CD300a- (pink) B cell subsets are distinguished. **(B)** Dot-bar graphs showing the median fluorescence intensity (MFI) of IgD, CD23, CD38, CD1c, CD25, CD86, HLA-DR, CD10, CD20 and CD21 in the indicated IgM^{hi}CD27+ B cell subsets. Each dot represents a donor. Statistical significance was determined using t-test or Wilcoxon matched-pairs signed rank test. *p< 0.05, **p< 0.01, ***p< 0.001 ****p< 0.0001, ns: not significant.

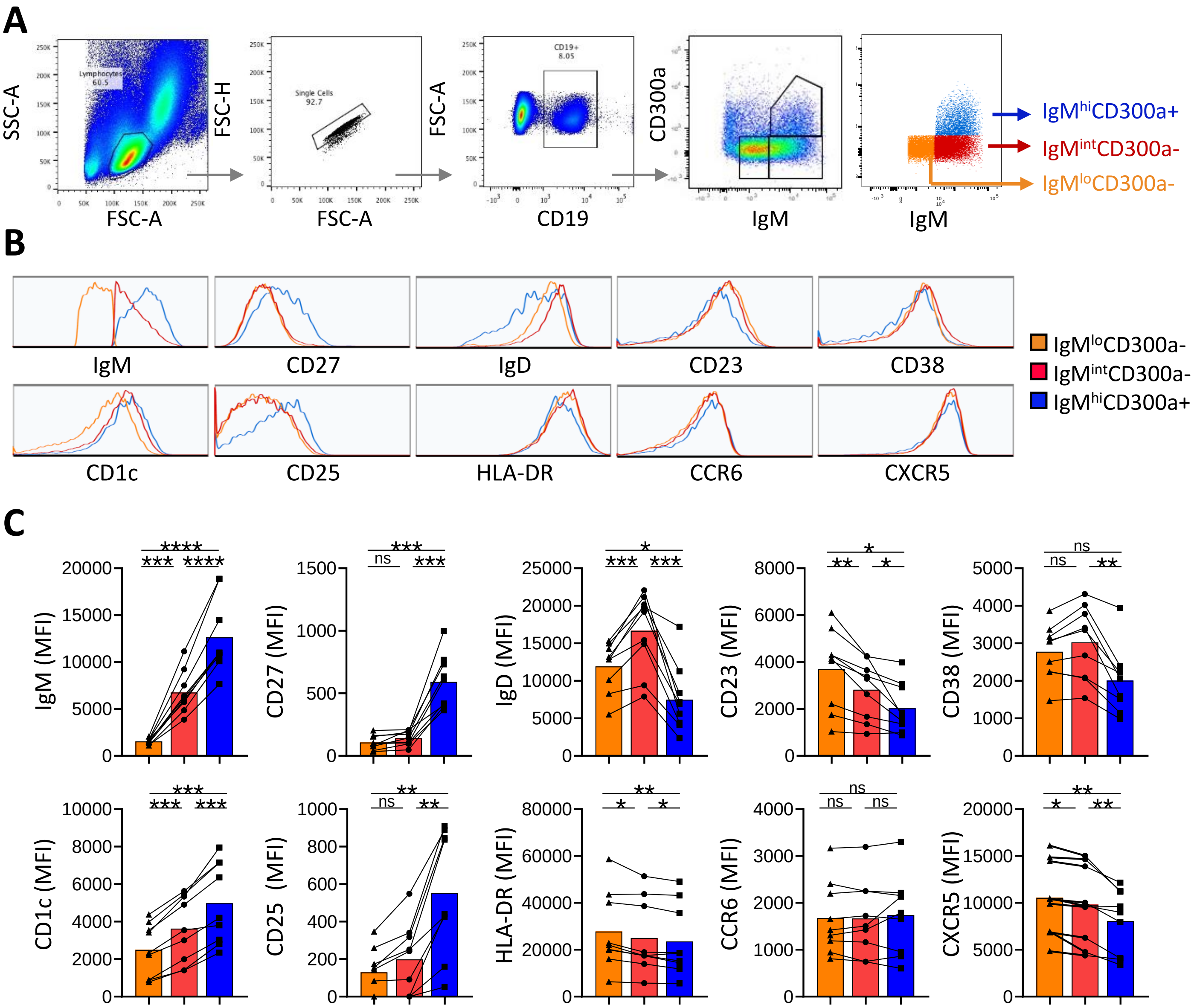

Supplementary Figure S2. Phenotypical characterization of IgM^{hi}CD300a^{+}, IgM^{int}CD300a^{-} and IgM^{lo}CD300a^{-} B cells from peripheral blood of healthy donors. (A) Pseudocolor plots of concatenated peripheral CD19^{+} B cells from healthy donors in which IgM^{lo}CD300a^{-} (orange), IgM^{int}CD300a^{-} (red) and IgM^{hi}CD300a^{+} (blue) B cell subsets are distinguished. **(B)** Histograms showing expression of IgM, CD27, IgD, CD23, CD38, CD1c, CD25, HLA-DR, CCR6 and CXCR5 in each B cell subset. **(C)** Dot-bar graphs showing the median fluorescence intensity (MFI) of IgM, CD27, IgD, CD23, CD38, CD1c, CD25, HLA-DR, CCR6 and CXCR5 in the indicated B cell subsets (n = 9). Each dot represents a donor. Means ± SEMs are shown. Statistical significance was determined by the Friedman test. *p< 0.05, **p < 0.01, ***p < 0.001, ****p < 0.0001, ns: not significant.

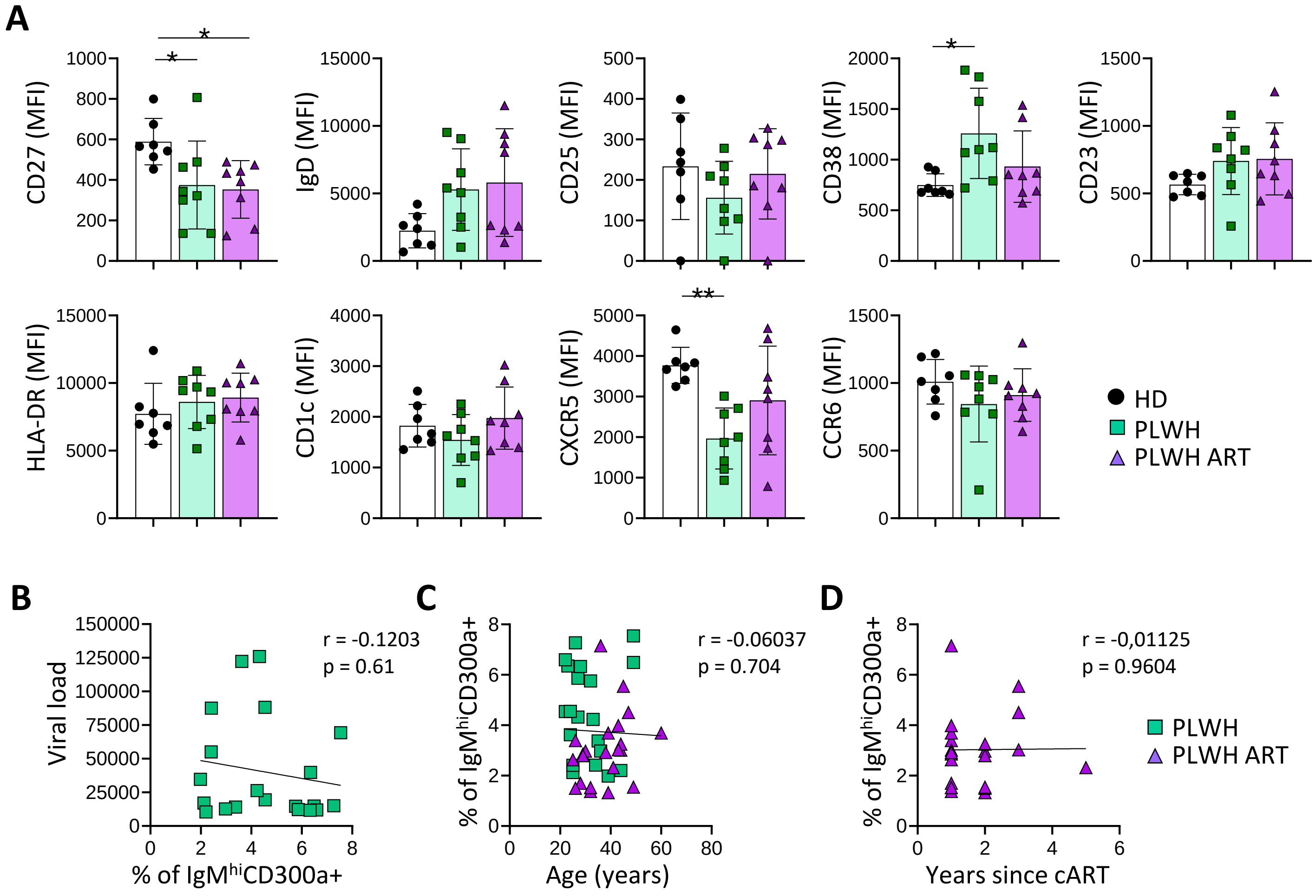

Supplementary Figure S3. (A) Dot-bar graphs showing the median fluorescence intensity (MFI) of CD27, IgD, CD25, CD38, CD23, HLA-DR, CD1c, CXCR5 and CCR6 in IgM^{hi}CD300a^{+} B cells from healthy donors (HD, white) ART naïve PLWH (PLWH, green) and PLWH under ART (PLWH ART, purple). Graphs showing the correlation of percentage of IgM^{hi}CD300a^{+} to the months viral load **(B)**, age in years **(C)** and years since ART **(D)** in ART naïve PLWH (PLWH, green squares) and PLWH under ART (PLWH ART, purple triangles). Kruskal-Wallis test (A) and Spearman (B, D) and Pearson (C) correlation tests were used.
